# Survival pattern of metastatic renal cell carcinoma patients according to WHO/ISUP grade: a long-term multi-institutional study

**DOI:** 10.1038/s41598-024-54052-6

**Published:** 2024-02-27

**Authors:** Joongwon Choi, Seokhwan Bang, Jungyo Suh, Chang Il Choi, Wan Song, Hyeong Dong Yuk, Chan Ho Lee, Minyong Kang, Seol Ho Choo, Jung Kwon Kim, Hyung Ho Lee, Jung Ki Jo, Eu Chang Hwang, Chang Wook Jeong, Young Hwii Ko, Jae Young Park, Cheryn Song, Seong Il Seo, Jinsoo Chung, Cheol Kwak, Sung-Hoo Hong

**Affiliations:** 1 Department of Urology, Chung-Ang University Gwangmyeong Hospital, Chung-Ang University College of Medicine, Gwangmyeong, Republic of Korea; 2 Department of Urology, Seoul St. Mary’s Hospital, The Catholic University of Korea, 222 Banpo-daero, Seocho-gu, Seoul, 06591 Republic of Korea; 3 Department of Urology, Asan Medical Center, University of Ulsan College of Medicine, Seoul, Republic of Korea; 4 Department of Urology, Hallym University Dongtan Sacred Heart Hospital, Hallym University School of Medicine, Hwaseong, Republic of Korea; 5 Department of Urology, Samsung Medical Center, Sungkyunkwan University School of Medicine, Seoul, Republic of Korea; 6 Department of Urology, Seoul National University College of Medicine, Seoul, Republic of Korea; 7 Department of Urology, Inje University Busan Paik Hospital, Inje University College of Medicine, Busan, Republic of Korea; 8 Department of Urology, Ajou University Hospital, Ajou University School of Medicine, Suwon, Republic of Korea; 9 Department of Urology, Seoul National University Bundang Hospital, Seoul National University College of Medicine, Seongnam, Republic of Korea; 10 Department of Urology, Center for Urologic Cancer, National Cancer Center, Goyang, Republic of Korea; 11 Department of Urology, Hanyang University Hospital, Hanyang University College of Medicine, Seoul, Republic of Korea; 12 Department of Urology, Chonnam National University Medical School, Gwangju, Jeonnam Republic of Korea; 13 Department of Urology, Yeungnam University College of Medicine, Daegu, Republic of Korea; 14 Department of Urology, Korea University Ansan Hospital, Korea University College of Medicine, Ansan, Republic of Korea

**Keywords:** Cancer, Oncology, Risk factors, Urology

## Abstract

The World Health Organization/International Society of Urological Pathology (WHO/ISUP) grading of renal cell carcinoma (RCC) is classified from grade 1–4, regardless of subtype. The National Comprehensive Cancer Network (NCCN) guidelines (2022) state that if there is an adverse pathological feature, such as grade 3 or higher RCC in stage 1 patients, more rigorous follow-up imaging is recommended. However, the RCC guidelines do not provide specific treatment or follow-up policies by tumor grade. Therefore, this study attempted to find out whether tumor grade affects survival rates in patients with metastatic RCC. The Korean Renal Cancer Study Group (KRoCS) database includes 3108 patients diagnosed with metastatic RCC between September 1992 and February 2017, with treatment methods, progression, and survival data collected from 11 tertiary hospitals. To obtain information on survival rates or causes of death, we utilized the Korea National Statistical Office database and institutional medical records. Data were accessed for research purpose on June, 2023. We then reviewed these sources to gather comprehensive and reliable data on the outcomes of our study cohort. This database was retrospectively analyzed, and out of 3108 metastatic RCC patients, 911 had been identified as WHO/ISUP grade. Grades were classified into either a low-grade (WHO/ISUP grade 1–2) or a high-grade group (WHO/ISUP grade 3–4). The patients were then analyzed related to progression and overall survival (OS). In metastatic clear cell RCC patients, the 1-year OS rate was 69.4% and the median OS was 17.0 months (15.5–18.5) followed up to 203.6 months. When comparing the patient groups, 119 low-grade and 873 high-grade cases were identified. No baseline difference was observed between the two groups, except that the high-grade group had a higher ECOG 1 ratio of 50.4% compared with 34.5% for the low-grade group (*p* = 0.009). There was a significant difference in OS between high-grade and low-grade groups. OS was 16.0 months (14.6–17.4) in the high-grade group and 28.0 months (21.1–34.9) in the low-grade group (*p* < 0.001). However, there was no difference in progression-free survival (PFS) rates with 9.0 months (8.0–10.0) for the high-grade group and 10.0 months (6.8–13.2) for the low-grade group (*p* = 0.377) in first-line treatment. In multivariable analysis, WHO/ISUP grade was a risk factor (HR = 1.511[1.135–2.013], *p* = 0.005) that influenced the OS. In conclusion, WHO/ISUP grade is a major data source that can be used as a ubiquitous marker of metastatic RCC in pre-IO era. Depending on whether the RCC is high or low grade, the follow-up schedule will need to be tailored according to grade, with higher-grade patients needing more active treatment as it can not only affect the OS in the previously known localized/locoregional recurrence but also the metastatic RCC patient.

## Introduction

Renal cell carcinoma (RCC) is a common malignancy of the urinary tract. In the United States, 81,000 new cases of RCC were diagnosed in 2023, and there were 14,890 related deaths^1^.

RCC’s grading system has been used as a prognostic factor for nearly 100 years. Although there are many grading systems, the Fuhrman grade was first used in 1982 after it was first reported that there was a difference in prognosis depending on nuclear size and cell outline^2^. The Fuhrman system was later replaced by the World Health Organization/International Society of Urological Pathology (WHO/ISUP) grading system in 2016. RCC’s WHO/ISUP grade is classified from grade 1 to grade 4, regardless of subtype. The grade is determined mainly by the shape of nucleoli, with nuclear pleomorphism, tumor giant cells, and rhabdoid or sarcomatoid differentiation also present in grade 4^3^.

With the development of advanced imaging techniques such as high-resolution CT/MRI, early detection of small RCC is increasing^4^. However, approximately 30% of patients with localized RCC eventually progress to disease recurrence or distant metastasis. Furthermore, 15–20% of RCC patients present with metastasis at the initial diagnosis^5^.

The National Comprehensive Cancer Network (NCCN) Guidelines (2022)^6^ state that if there is an adverse pathological feature such as a high grade of grade 3 or higher in stage 1 patients, more rigorous follow-up imaging is recommended. However, it does not include any specific information or treatment plan for metastatic RCC thus far. The importance of grading tumors is emphasized most clearly in the “follow-up after surgery” section of the American Urological Association guidelines^7^. For pT1 tumors, which include tumors up to 7 cm in size, the tumors are divided into low/intermediate risk based on grade 1–2 or 3–4. Depending on the risk level, different follow-up schedules are recommended. For low-risk tumors, follow-up after one year of surgery is recommended, while annual follow-ups are suggested recommended for all risk levels three years after surgery. No distinction is made based on the grade for tumors classified as pT2 or higher.

Metastatic RCC contains several subgroups that differ significantly in terms of clinical characteristics and prognoses^8^. However, if there is a prognosticator that helps predict prognosis, it can guide patient treatment. Therefore, this study attempted to investigate whether grade affects survival in patients with metastatic RCC in a large-volume database registry.

## Materials and methods

The Korean Renal Cancer Study Group (KRoCS) was created in 2013 and comprises data from 11 university hospitals in Korea^9^. Since March 2014, a web-based metastatic kidney cancer database system for RCC has been established^10^. The database was named KRoCS database, and it contained the 3108 patients diagnosed with metastatic RCC from September 1992 to February 2017, along with the treatment methods, progression, and survival data collected from the 11 tertiary hospitals. It also contains data on what primary, secondary, and tertiary treatments the RCC patients received. Also, the survival status was updated in July 2018 with no patients enrolled from February 2017. All institutions were approved by their institutional review board committees before being enrolled in the database. Due to the retrospective nature of the database, Institutional Review Board of Seoul National University Bundang Hospital, and has been approved by all relevant institutions (B-1902-522-101), waived the need of obtaining informed consent. We have conducted an IRB review for this research topic, the Institutional Review Board of Chung-Ang University Gwangmyeoung Hospital approved this study (approval number: 2304-076-039). This study was conducted according to the ethical standards recommended by the 1964 Declaration of Helsinki and its later amendments.

Data were accessed for research purpose on June, 2023, and we retrospectively reviewed 3,108 metastatic RCC patients, with 911 patients confirmed as having been given WHO/ISUP grade in this database. We excluded 2197 patients from the current study because they either lacked survival data or grade records. To obtain information on survival and cause of death, we utilized the Korea National Statistical Office database along with institutional medical records. We reviewed these sources to gather comprehensive and reliable data on the outcomes of our study cohort. Patients were classified into either a low-grade (grade 1–2) or a high grade (grade 3–4), then analyzed related to progression and overall survival (OS). Progression was defined according to radiographic criteria based on RECIST (Response Evaluation Criteria in Solid Tumors) ver 1.1^11^.

In Tables 1, 2, and 3, the comparison between the two groups was conducted using Student’s t-test to compare means, and Fisher’s exact test was employed for the comparison of two categorical variables. For the comparison of overall survival and progression-free survival, Kaplan–Meier survival analysis and the log-rank test were employed. Multivariate Cox-regression model was used to identify overall and PFS predictors in Table 4 and 5. Statistical significance was set at *p* < 0.05. The SPSS software package (version 27.0; Statistical Package for Social Sciences, Chicago, IL, USA) and MedCalc (version 20; MedCalc Software, Ostend, Belgium) was used for all statistical analyses. All data used in the statistics has been provided in the supplementary material.

**Table 1:** Baseline characteristics.

	Low grade		High grade	*p*
	(N = 119)		(N = 873)	
Age	59.2 ± 10.9		57.0 ± 11.6	0.052
Follow up (mo)	38.38 ± 38.69		26.6 ± 27.1	0.935
Body weight (kg)	64.0 ± 9.2		73.8 ± 291.3	0.336
Height (cm)	164.4 ± 8.3		165.8 ± 8.1	0.092
Body mass index (BMI)	23.6 ± 2.8		23.2 ± 3.4	0.091
Smoking				
Non-smoker	66 (55.5%)		480 (55.2%)	0.402
ex-smoker	27 (22.7%)		220 (25.3%)	
Current smoker	19 (16.0%)		144 (16.6%)	
Unknown	7 (5.9%)		26 (3.0%)	
Heng risk group				
Favorable	1 (0.8%)		8 (0.9%)	0.079
Intermediate	73 (61.3%)		468 (53.6%)	
Poor	43 (36.1%)		394 (45.1%)	
Unknown	2 (1.7%)		3 (0.3%)	
ECOG performance status				
0	66 (55.5%)		354 (40.5%)	0.009
1	41 (34.5%)		440 (50.4%)	
2	7 (5.9%)		39 (4.5%)	
Unknown	5 (4.2%)		40 (4.6%)	
Diabetes	30 (25.2%)		153 (17.5%)	0.057
Hypertension	42 (35.3%)		354 (40.5%)	0.318
Chronic kidney disease				
No dialysis	115 (96.6%)		844 (96.7%)	0.862
Dialysis or transplantation	4 (3.4%)		29 (3.3%)	
Cerebrovascular accident	0 (0.0%)		15 (1.7%)	0.298
Clinical T stage				
cT1	31 (26.1%)		199 (22.8%)	0.469
cT2	20 (16.8%)		194 (22.2%)	
cT3	40 (33.6%)		310 (35.4%)	
cT4	7 (5.9%)		74 (8.5%)	
cTx	21 (17.6%)		96 (11.0%)	
Clinical N stage				
cN0	90 (75.6%)		556 (63.6%)	0.057
cN1	25 (21.0%)		287 (32.9%)	
cNx	4 (3.4%)		30 (3.4%)	
Clinical & pathologic M stage				
cM0	11 (9.2%)		112 (12.8%)	0.510
cpM1	108 (90.8%)		761 (87.2%)

**Table 2:** Pathologic status of metastatic RCC patients.

	Low grade	High grade	*p*
	(N = 119)	(N = 873)	
Pathologic T stage			
pT1a	15 (12.6%)	44 (5.0%)	< 0.001
pT1b	28 (23.5%)	90 (10.3%)	
pT2a	13 (10.9%)	85 (9.7%)	
pT2b	5 (4.2%)	38 (4.4%)	
pT3a	35 (29.4%)	469 (53.7%)	
pT3b	6 (5.0%)	71 (8.1%)	
pT3c	0 (0.0%)	4 (0.5%)	
pT4	12 (10.1%)	61 (7.0%)	
pTx	5 (4.2%)	11 (1.3%)	
WHO/ISUP nuclear grade			
Grade 1	5 (4.2%)	0 (0.0%)	< 0.001
Grade 2	114 (95.8%)	0 (0.0%)	
Grade 3	0 (0.0%)	508 (58.2%)	
Grade 4	0 (0.0%)	365 (41.8%)	
RCC type			
Clear cell	110 (93.2%)	772 (88.4%)	0.336
Papillary	7 (5.9%)	42 (4.8%)	
Chromophobe	0 (0.0%)	9 (1.0%)	
Collecting duct	0 (0.0%)	10 (1.1%)	
Unclassified	0 (0.0%)	17 (1.9%)	
xp11.2 transposition	0 (0.0%)	13 (1.5%)	
Others	1 (0.8%)	10 (1.2%)	
Sarcomatoid component	8 (6.7%)	190 (21.8%)	< 0.001
Resection margin			
Negative	117 (98.3%)	843 (96.6%)	0.459
Positive	2 (1.7%)	30 (3.4%)	

RCC: renal cell carcinoma.

**Table 3:** Treatment type & survival data.

	Low grade			High grade	*p*
	(N = 119)			(N = 873)	
First-line systemic treatment					0.290
cytokines	24 (22.6%)			136 (16.4%)	
TKIs	78 (73.6%)			633 (76.5%)	
mTOR inhibitors	3 (2.8%)			47 (5.7%)	
others	1 (0.9%)			11 (1.3%)	
First-line cytokines type					
IFN + chemo	15 (62.5%)			51 (37.5%)	0.143
IL-2 + chemo	1 (4.2%)			10 (7.4%)	
IL-2 + IFN + chemo	8 (33.3%)			75 (55.1%)	
First-line TKI type					
sunitinib	52 (66.7%)			388 (61.3%)	0.513
sorafenib	14 (17.9%)			88 (13.9%)	
pazopanib	12 (15.4%)			147 (23.2%)	
axitinib	0 (0.0%)			3 (0.5%)	
bevacizumab + IFN	0 (0.0%)			5 (0.8%)	
others	0 (0.0%)			2 (0.3%)	
First-line mTOR type					
everolimus	2 (66.7%)			14 (29.8%)	0.491
temsirolimus	1 (33.3%)			33 (70.2%)	
First-line PFS (mo)	10.7 ± 14.0			9.1 ± 13.9	0.277
Second-line systemic treatment					0.300
cytokines	5 (9.4%)			18 (3.9%)	
TKIs	18 (34.0%)			170 (37.3%)	
mTOR inhibitors	27 (50.9%)			249 (54.6%)	
others	3 (5.7%)			19 (4.2%)	
Second-line cytokines type					
IFN + chemo	2 (40.0%)			7 (38.9%)	0.260
IL-2 + chemo	2 (40.0%)			4 (22.2%)	
IL-2 + IFN + chemo	1 (20.0%)			7 (38.9%)	
Second-line TKI type					
sunitinib	8 (44.4%)			79 (45.9%)	0.698
sorafenib	5 (27.8%)			54 (31.4%)	
pazopanib	5 (27.8%)			29 (16.9%)	
axitinib	0 (0.0%)			7 (4.1%)	
others	0 (0.0%)			3 (1.7%)	
Second-line mTOR type					
everolimus	26 (96.3%)			238 (96.4%)	1.000
temsirolimus	1 (3.7%)			9 (3.6%)	
Second-line PFS (mo)	6.7 ± 10.7			6.6 ± 10.5	0.970
Third-line systemic treatment					
cytokines	1 (6.7%)			24 (13.6%)	0.170
TKIs	4 (26.7%)			69 (39.2%)	
mTOR inhibitors	10 (66.7%)			68 (38.6%)	
others	0 (0.0%)			15 (8.5%)	
Third-line cytokines type					
IFN + chemo	1 (100.0%)			15 (62.5%)	0.746
IL-2 + chemo	0 (0.0%)			6 (25.0%)	
IL-2 + IFN + chemo	0 (0.0%)			3 (12.5%)	
Third-line TKI type					
sunitinib	3 (75.0%)			12 (17.4%)	0.081
sorafenib	0 (0.0%)			32 (46.4%)	
pazopanib	1 (25.0%)			19 (27.5%)	
axitinib	0 (0.0%)			4 (5.8%)	
others	0 (0.0%)			2 (2.9%)	
Third-line mTOR type					
everolimus	10 (100.0%)			63 (92.6%)	0.845
temsirolimus	0 (0.0%)			5 (7.4%)	
Third-line PFS (mo)	4.8 ± 6.2			5.8 ± 10.2	0.576

*TKIs* tyrosine kinase inhibitors; *IFN* interferon; *chemo* chemotherapy; *PFS* progression-free survival.

**Table 4:** Predictive Factors including grade for overall survival based on multivariate regression analysis.

	Adjusted hazard ratio	95% CI			*p*
Age	0.999	(0.991	–	1.007)	0.828
WHO/ISUP grade					
Low	1.000				
High	1.511	(1.135	–	2.013)	0.005
smoking					
Non-smoker	1.000				
Ex-smoker	1.229	(1.005	–	1.502)	0.045
Current smoker	0.937	(0.744	–	1.180)	0.581
Unknown	1.741	(0.538	–	5.628)	0.355
MSKCC					
Favorable	1.000				
Intermediate	4.478	(0.613	–	32.698)	0.139
Poor	5.104	(0.681	–	38.242)	0.113
ECOG					
0	1.000				
1	0.861	(0.705	–	1.051)	0.142
2	1.096	(0.740	–	1.623)	0.646
Unknown	0.824	(0.470	–	1.444)	0.498
DM	0.903	(0.723	–	1.128)	0.368
HTN	0.968	(0.812	–	1.155)	0.721
CKD	1.068	(0.695	–	1.639)	0.765
CVA	1.105	(0.484	–	2.527)	0.812
RCC type					
Clear cell	1.000				
Papillary	1.586	(1.099	–	2.289)	0.014
Chromophobe	0.709	(0.253	–	1.983)	0.512
Collecting duct	1.462	(0.627	–	3.408)	0.380
Unclassified	0.660	(0.357	–	1.221)	0.186
xp11.2 transposition	1.227	(0.548	–	2.746)	0.618
Others	1.491	(0.466	–	4.770)	0.501
Unknown	1.395	(0.492	–	3.949)	0.531
Sarcomatoid	1.617	(1.303	–	2.008)	< 0.001
Margin					
Negative	1.000				
Positive	1.828	(1.147	–	2.913)	0.011

RCC: renal cell carcinoma.

**Table 5:** Predictive Factors including grade for progression-free survival based on multivariate regression analysis.

	Adjusted hazard ratio	95% CI			*p*
Age	1.003	(0.996	–	1.011)	0.425
WHO/ISUP grade					
Low	1.000				
High	1.072	(0.845	–	1.358)	0.567
Smoking					0.317
Non-smoker	1.000				
Ex-smoker	1.981	(0.488	–	8.043)	0.339
Current smoker	2.215	(0.544	–	9.017)	0.267
Unknown	1.803	(0.441	–	7.365)	0.412
MSKCC					0.900
Favorable	1.000				
Intermediate	0.670	(0.165	–	2.719)	0.575
Poor	0.658	(0.159	–	2.728)	0.564
ECOG					
0	1.000				
1	0.999	(0.843	–	1.184)	0.991
2	1.180	(0.819	–	1.701)	0.374
Unknown	1.172	(0.651	–	2.110)	0.596
DM	1.077	(0.890	–	1.302)	0.446
HTN	0.958	(0.821	–	1.119)	0.590
CKD	1.002	(0.680	–	1.475)	0.992
CVA	0.873	(0.500	–	1.527)	0.635
RCC type					
Clear cell	1.000				
Papillary	2.046	(1.388	–	3.014)	< 0.001
Chromophobe	0.961	(0.417	–	2.214)	0.925
Collecting duct	1.793	(0.870	–	3.693)	0.113
Unclassified	0.814	(0.457	–	1.449)	0.483
xp11.2 transposition	0.980	(0.522	–	1.840)	0.950
Others	1.203	(0.488	–	2.963)	0.689
Unknown	0.778	(0.280	–	2.160)	0.630
Sarcomatoid	1.446	(1.205	–	1.736)	< 0.001
Margin					
Negative	1.000				
Positive	1.402	(0.916	–	2.146)	0.120

*RCC* renal cell carcinoma.

## Results

Baseline characteristics and collected data are shown in Table 1. When comparing the patient groups, 119 low grades and 873 high grades were identified, with a median follow-up of 18.9 months (IQR 8.4–36.9). There was no baseline statistical difference between the two groups, except that the high-grade group had a higher ECOG 1 ratio of 50.4% compared with 34.5% (*p* = 0.009).

Detailed pathologic status is shown in Table 2. In both groups, the radical nephrectomy implementation rate was approximately 95% (low 95.8% vs. high 96.1%, *p* = 0.874), and in most cases was a clear cell type (93.2% vs. 88.4%, *p* = 0.0336). Additionally, the sarcomatoid ratio was significantly higher in the high grade (6.7% vs. 21.8%, *p* < 0.001).

**Figure 1:**
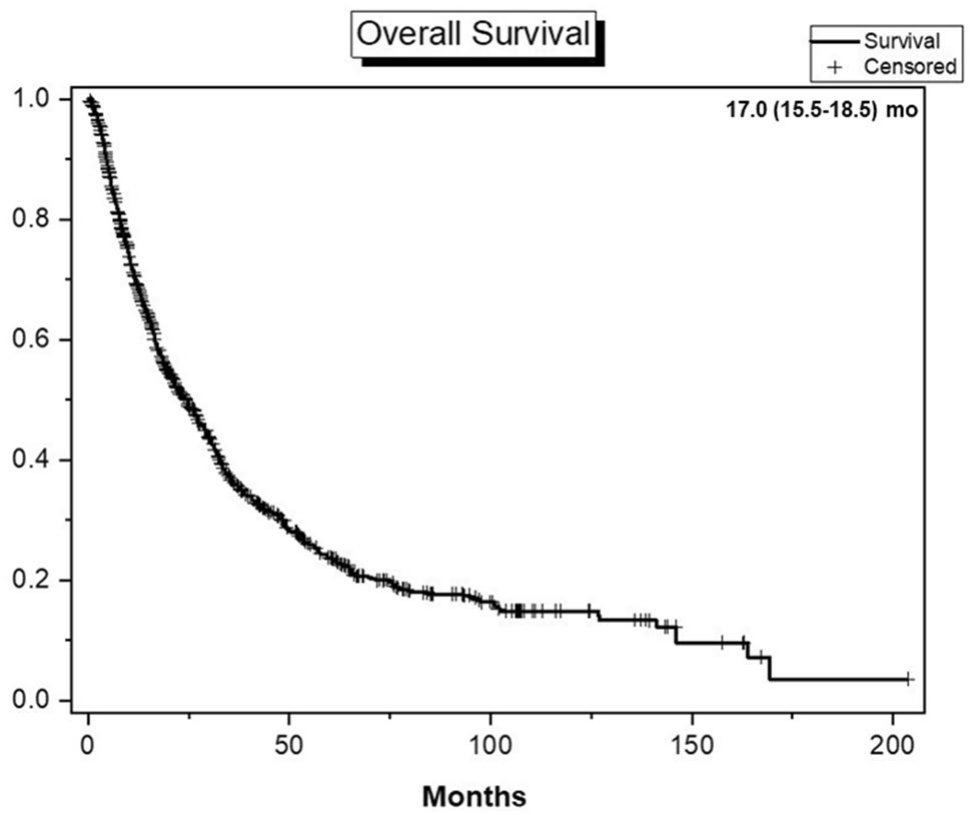
Overall survival of metastatic RCC patient. Overall survival graph of 911 metastatic RCC patients from the KRoCS (Korean Renal Cancer Study Group) database from September 1992 to February 2017.

Over a period of 25 years, various drugs such as cytokines, tyrosine kinase inhibitors (TKIs), and mTOR inhibitors have been used for treatment of metastatic RCC. TKI was mainly used as the first-line treatment (73.6% and 76.5%, *p* = 0.290), and there was no statistical difference in the treatment applied to the two groups (Table 3). In total, the 1-yr OS was 69.4% and the median OS was 17.0 months (15.5–18.5), with follow-up of up to 203.6 months (Fig. 1).

**Figure 2:**
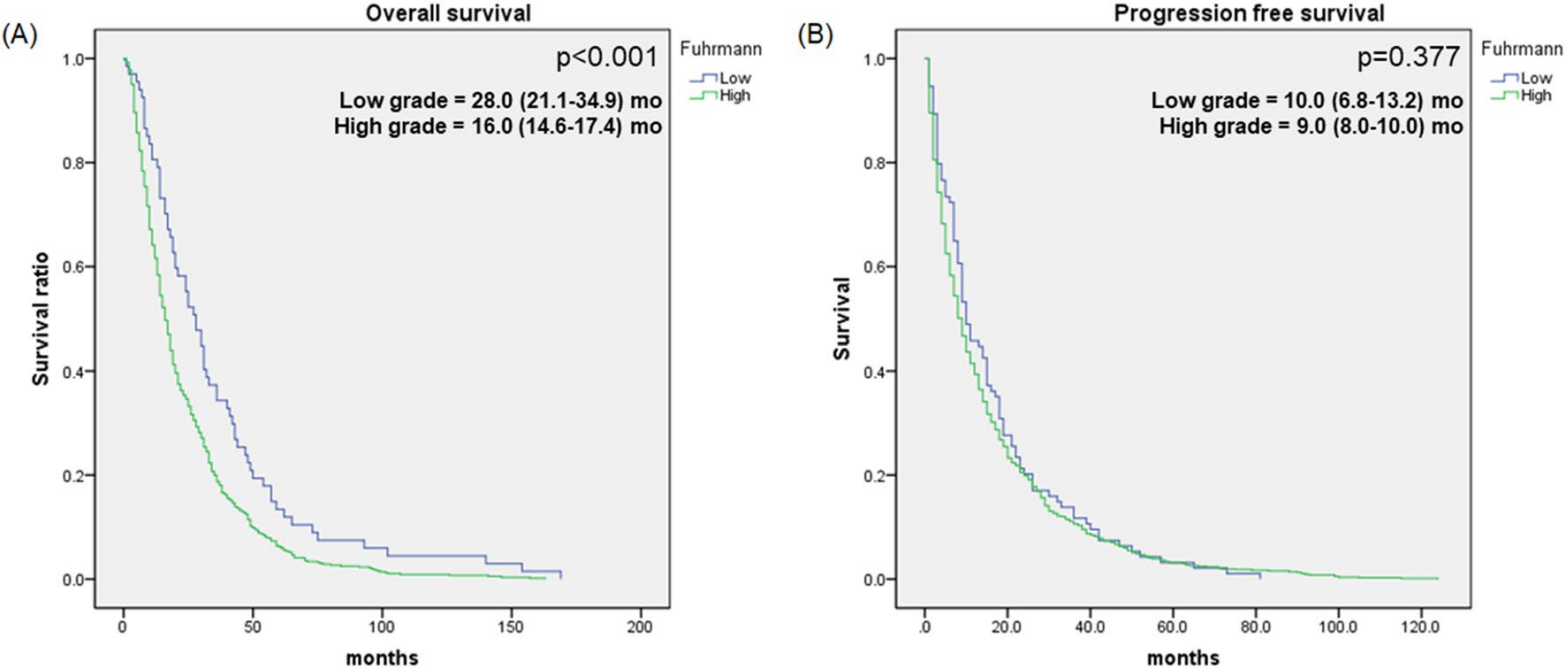
Overall survival and progression-free survival of metastatic RCC patients by grade. (**A**) Overall survival graph of metastatic RCC patients with low (blue line) and high (green line) grades (total n = 911, *p* < 0.001). (**B**) Progression-free survival graph of metastatic RCC patients with low (blue line) and high (green line) grades in first-line treatment (total n = 911, *p* = 0.377).

There was a significant difference in OS between the high-grade and low-grade groups (Fig. 2). The OS was 16.0 months (14.6–17.4) for the high-grade group and 28.0 months (21.1–34.9) for the low-grade group (*p* < 0.001). However, there was no significant difference in progression-free survival (PFS), with 9.0 months (8.0–10.0) for the high-grade group and 10.0 months (6.8–13.2) for the low-grade group (*p* = 0.377). In a multivariable analysis for OS (Table 4), WHO/ISUP grade (HR = 1.511[1.135–2.013], *p* = 0.005) influenced OS with patients who were ex-smokers (HR = 1.229, *p* = 0.045), with papillary RCC (HR = 1.586, *p* = 0.014), sarcomatoid component (HR = 1.617, *p* < 0.001) and margin status (HR = 1.828). According to the multivariable analysis related to progression-free survival (Table 5), papillary RCC (HR = 2.046, *p* < 0.001) and sarcomatoid component (HR = 1.446, *p* < 0.001) were both risk factors for cancer progression in first-line treatment.

Results excluding Chromophobe RCC are provided in Supplementary Material S2.

**Figure 3:**
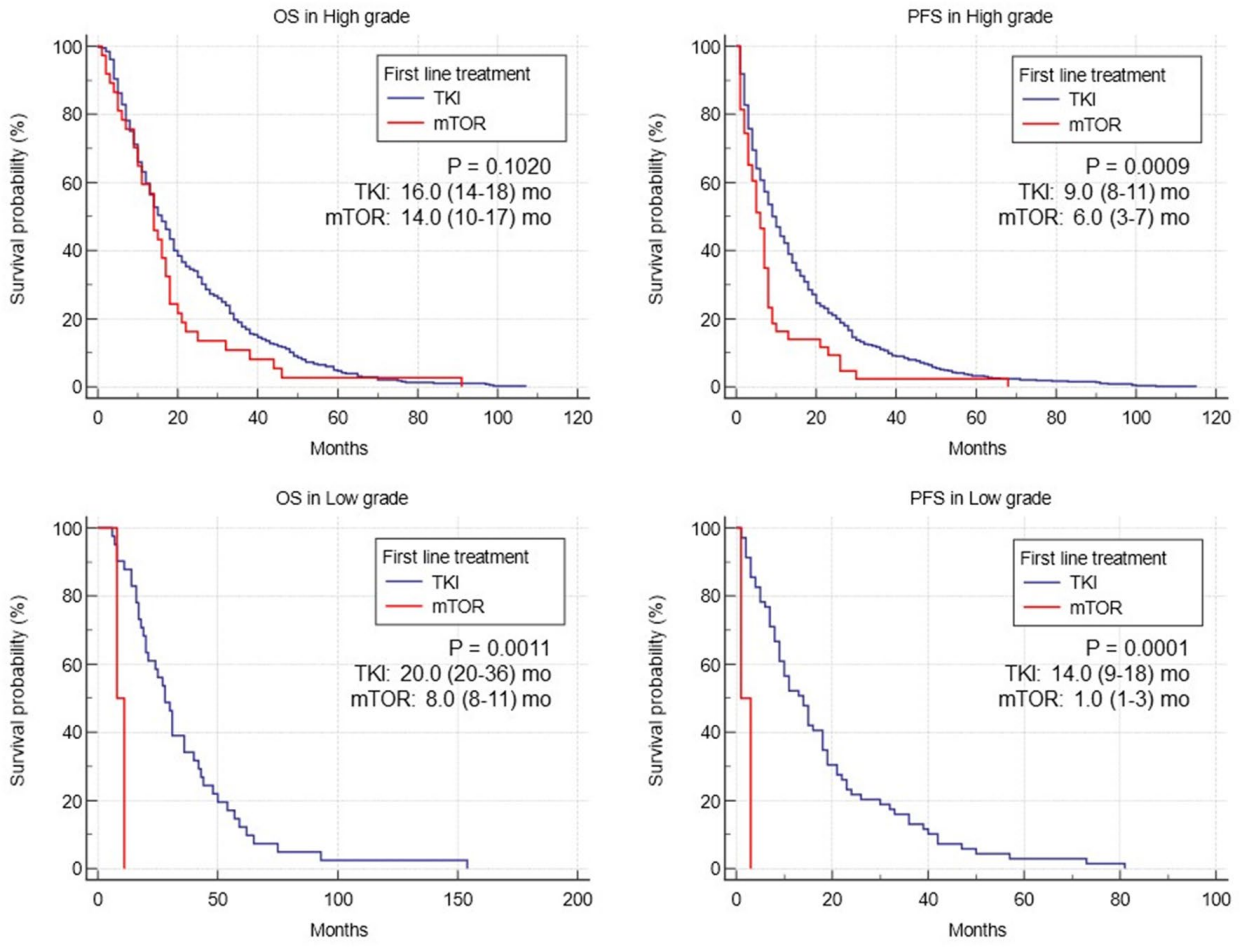
Overall survival and progression-free survival by grade and treatment type (TKI or mTOR). Overall survival and progression-free survival graph of metastatic RCC patients with first-line TKI (blue) and mTOR (red) treatment by grade.

Furthermore, we investigated whether there is a difference in the effects of TKI and mTOR, representative treatments for metastatic RCC in pre-IO era, between high and low grades (Fig. 3). The results indicated a grade-dependent correlation, where TKI as a first-line treatment led to extended OS and PFS (all *p* < 0.05). Particularly in low-grade cases, the impact of TKI was more pronounced (all *p* < 0.01).

**Figure 4:**
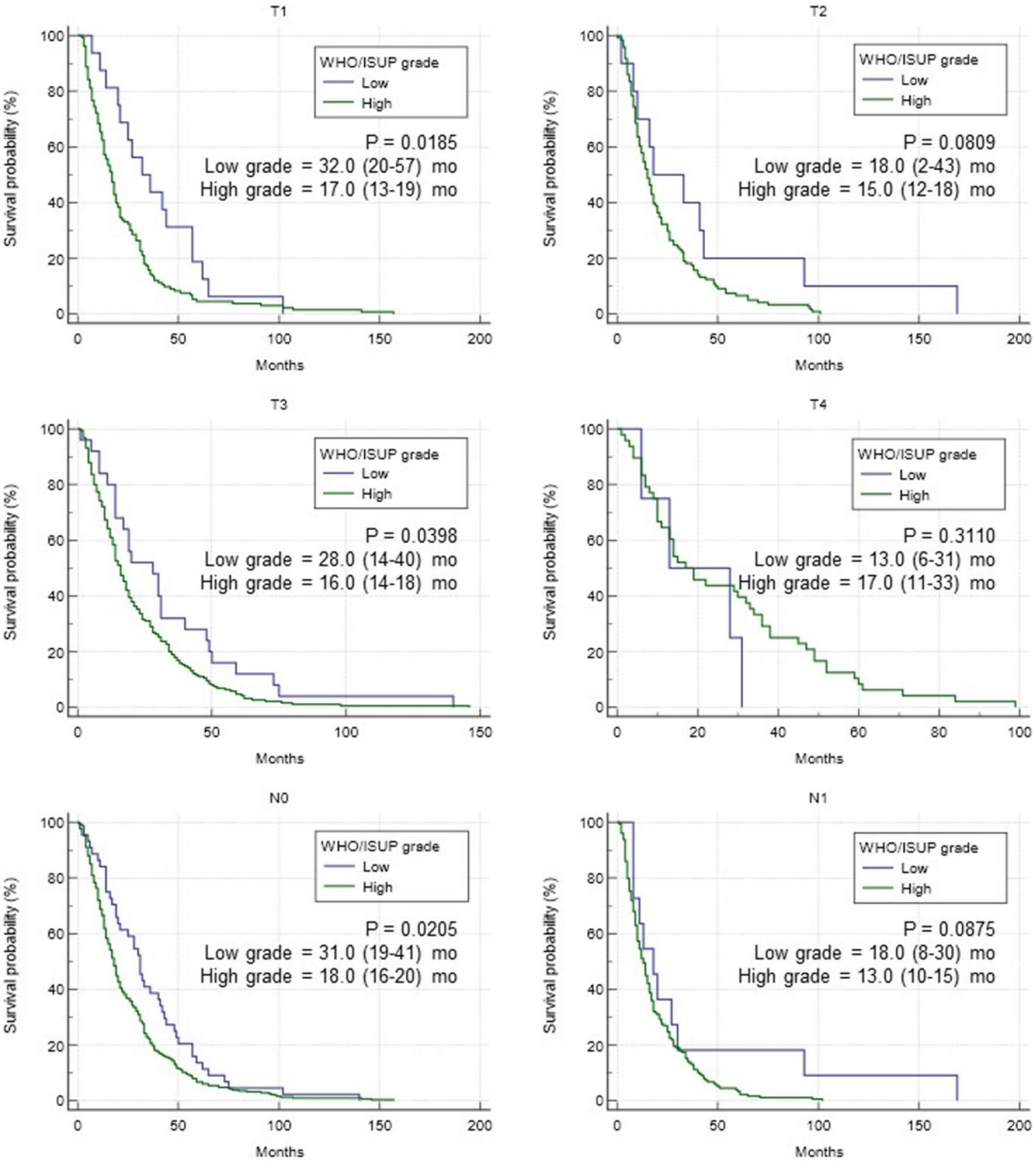
Overall survival by grade with T and N stage. Overall survival of metastatic RCC patients was classified by T and N stage.

Lastly, to further stratify the impact of grade, we conducted OS analysis based on each T and N stage (Fig. 4). As a result, in T1, T3, and N0 stages, a statistically significant prolongation of OS was observed in the low-grade group (all *p* < 0.05). However, in T2, T4, and N1 stages, relatively higher stage, no significant difference was observed between the two groups.

## Discussion

Our study revealed that there is approximately a one-year difference in OS depending on whether the RCC is high or low grade. This finding underscores the importance of considering tumor grade as a prognostic factor in the management of metastatic RCC. This result also indicates the potential value of considering grades in future follow-up schedules and observations.

Clear cell RCC is the predominant subtype of RCC, comprising approximately 80% of cases according to the World Health Organization (WHO) classification system. The other subtypes include papillary RCC, chromophobe RCC, collecting duct RCC, unclassified RCC, and Xp11.2 translocation RCC^10^. Metastatic RCC is a complex disease consisting of diverse subtypes, each with distinct morphological, genetic, clinical, and prognostic features^12^. These subgroups exhibit significant heterogeneity, making the accurate diagnosis and effective treatment of metastatic RCC challenging.

While there are relatively few studies that focus on the association between Fuhrman grades or WHO/ISUP grades and RCC, this is nonetheless a steadily emerging field. However, reports on this topic in metastatic RCC are scarce, and the value of tumor grading is not strongly emphasized in the guidelines of the National Comprehensive Cancer Network (NCCN), the European Association of Urology (EAU), or the AUA for the management of metastatic RCC.

A study similar to ours enrolled 266 patients with metastatic RCC who received treatment with TKIs^13^. They examined several serum biomarkers, including the neutrophil-to-lymphocyte ratio, and found that WHO/ISUP grade 3–4 increased the risk of metastatic RCC, with an HR of approximately 2.0. Their risk model revealed that there was a clear difference in OS based on the number of risk factors, with six risk factors indicating the highest risk.

In 2020, a nomogram study using the Surveillance, Epidemiology, and End Results (SEER) database was published^14^. The study enrolled 12,216 patients with metastatic RCC between 2010 and 2016 and used a training set of 1158 patients and a validation set of 1157 patients to develop the nomogram. Their multivariable analysis revealed that WHO/ISUP grade was a risk factor with the risk increasing with each grade. They assigned scores of 0 and 5 for WHO/ISUP grades 1–2, 20 for grade 3, and approximately 40 for grade 4 before calculating the total score to predict survival rates at one, three, and five years.

Generally, it is expected that low-grade tumors will have a better prognosis, while high-grade tumors may have a poorer prognosis. However, in our analyzed data, there are only cases of patients with low-grade tumors who developed metastasis. There is a possibility of selection bias within the analyzed patient group. Since our study includes only lower-grade cancer patients who have experienced metastasis, there is a higher likelihood of including a patient group with unfavorable conditions for metastasis, rather than representing the characteristics of the entire low-grade patient population. Furthermore, this could be a likely reason why the impact of grade appears relatively diminished in metastatic RCC. As a similar example, in the paper discussing late recurrence in patients with RCC, stating that T1a stage patients experience later recurrence more than T1b stage patients^15^. However, this phenomenon might not mean the actual truth that low-stage patients experience late recurrence more, but rather that high-stage patients experience more early recurrences, leading to a relatively lowerer proportion of high-stage patients in the late recurrence category.

A study on the risk factors for locoregional recurrence in patients who underwent radical nephrectomy^16^ focused on patients with T3–4 tumors in a non-metastatic setting. The results showed that locoregional recurrence was strongly associated with a sharp decline in five-year OS and that Fuhrman grade IV was a powerful risk factor for recurrence with an HR of 3.6 in multivariable analysis. This result indirectly suggests that Fuhrman grade IV may also impact OS.

Regarding grading of chromophobe RCC, there was no difference in OS among the three cell types (clear cell, papillary, chromophobe) for low-grade tumors (grades 1–2), but in high-grade tumors (grades 3–4), chromophobe RCC shows similar survival outcomes to low grade, while clear cell and papillary RCC have lower survival rates^17^. A recent study, therefore, has argued that the chromophobe tumor grade (CTG), consisting of three categories, should be used as a grading system^18^. Alternatively, Ohashi et al. have proposed a two-category grading system that only considers the presence of tumor necrosis or sarcomatoid component^19^. Regardless of RCC sub-classification, the sarcomatoid component has been identified as a prognostic factor for overall survival (HR = 1.617, *p* < 0.001) and PFS (HR = 1.446, *p* < 0.001). In our study, the high-grade cohort exhibited a 21.8% sarcomatoid component, while the low-grade cohort showed only 6.7%. Whether the sarcomatoid component and high grade are entirely independent factors is not fully understood, and sarcomatoid differentiation is also a characteristic of WHO/ISUP grade 4. Further research is necessary.

Although there is limited information on the mechanism of this grading phenomenon, a 2020 study suggested that as immunotherapy becomes more established as a standard treatment for RCC, the dysfunction of CD4 and CD8 T cells infiltrating tumor tissue is more pronounced in higher-grade tumors^20^. This indicates that immune responses may not function as effectively in high-grade RCC. The study examined 97 patients and found that in WHO/ISUP grades 3–4, CD4 and CD8 T cells were upregulated within cancer cells, while cytokine production was significantly lower. The results showed that while the cell ratio was higher in high-grade RCC, the proportion of granzyme B, which is associated with cytotoxic activity, was lower, indicating that effective immune responses did not occur.

Our study has some limitations that should be considered. First, the study cohort is highly heterogeneous due to the inclusion of patients with varying characteristics such as different first-line treatment agents, metastasis sites, and previous cytoreductive nephrectomy or metastasectomy statuses. However, we believe that grade could provide value as a ubiquitous marker for metastatic RCC in pre-IO era, further research on whether this holds true in the IO era would be highly valuable. Second, we did not perform a central pathology review, which may have resulted in some variability in the accuracy of our diagnosis. Third, our database includes the era of TKIs and does not include information on immune checkpoint inhibitors (IO) such as TKI + IO combinations, IO + IO combinations, and adjuvant IO, which are currently being actively studied. Our database is currently updated only until July 2018, and subsequent updates have been hindered by ongoing changes in IO treatment, compounded by restrictions on gatherings due to COVID-19. Therefore, new data on the impact of grades in the IO era is required and we are planning to update the database, including IO treatment. Fourth, this study has a retrospective design, there is a possibility of potential selection bias in our study. And while there were no deviations in data collection, the WHO/ISUP grade was only introduced in 2016 and was used interchangeably with the Fuhrman grade.

Despite the retrospective nature of this study, we believe that it holds significant value as it is based on long-term follow-up multicenter data obtained from a database^21^. The grading of RCC based only on cell morphology includes more than 11 categories according to the WHO classification^3^. Therefore, it may not be appropriate to uniformly classify the grade of RCC. However, even when considering RCC cell types without differentiation, as in our study, there was a significant difference in OS rates, suggesting that it has a meaningful role as a ubiquitous marker. Therefore, we consider our findings to be reliable and informative for future research in this field. It would also be valuable in comparison with the results of the IO era.

## Conclusion

In conclusion, WHO/ISUP grade is a major data source that can be used as a ubiquitous marker of metastatic RCC in pre-IO era. Depending on whether the RCC is high or low grade, the follow-up schedule will need to be tailored according to grade, with higher-grade patients needing more active treatment as it can not only affect the OS in the previously known localized/locoregional recurrence but also the metastatic RCC patient.

### Supplementary Information

Supplementary Information 1.

Supplementary Information 2.

## Data Availability

The authors declare that all data generated or analysed during this study are included in the Source Data file provided in the Supplementary Information files S1.
