# Supplementary material for: Survival pattern of metastatic renal cell carcinoma patients according to WHO/ISUP grade: a long-term multi-institutional study
Source: Sci Rep. 2024 Feb 27;14:4740. doi: 10.1038/s41598-024-54052-6 (PMC10899595; doi:10.1038/s41598-024-54052-6)
Supplement: Supplementary file 2 — Supplementary Information 2. [file 41598_2024_54052_MOESM2_ESM.pptx]

## Slide 1
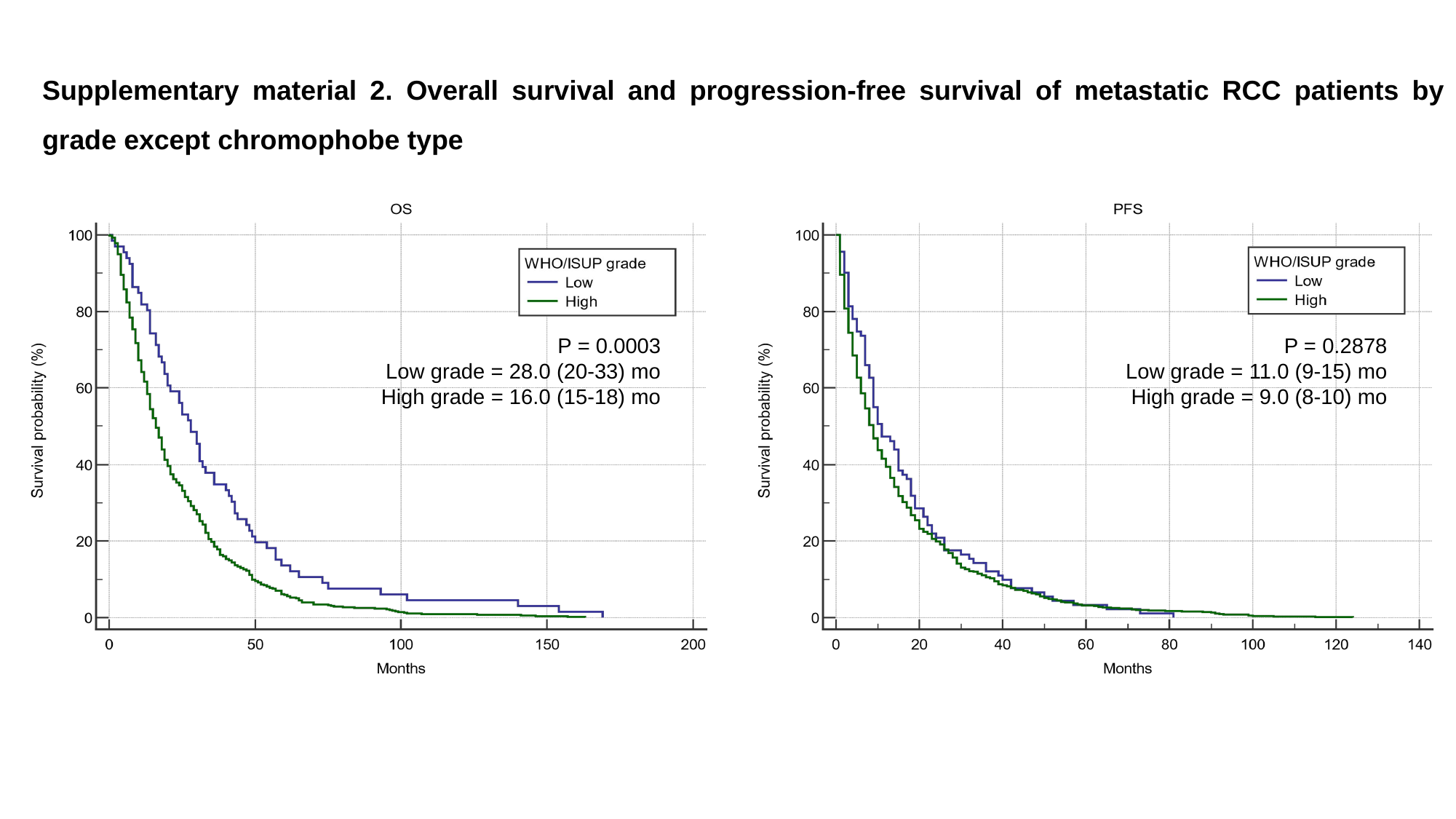

Supplementary material 2. Overall survival and progression-free survival of metastatic RCC patients by grade except chromophobe type
P = 0.0003
Low grade = 28.0 (20-33) mo
High grade = 16.0 (15-18) mo
P = 0.2878
Low grade = 11.0 (9-15) mo
High grade = 9.0 (8-10) mo
